# Int Braz J Urol Annual Report – 2012 – 2019

**DOI:** 10.1590/S1677-5538.IBJU.2019.06.01

**Published:** 2019-12-17

**Affiliations:** 1 Disciplina de Urologia, Faculdade de Medicina do ABC, Santo André, SP, Brasil

The Brazilian Society of Urology (*Sociedade Brasileira de Urología* (SBU)) was founded in 1926 and in 1974 was launched the Brazilian Journal of Urology (*Jornal Brasileiro de Urologia*), a journal dedicated to publish the research from the Brazilian Urological community.

Without missing one issue the Jornal Brasileiro de Urologia has evolved to the International Brazilian Journal of Urology (IBJU), now a journal to where 2130 authors of 542 institutions from 62 countries have submitted 891 papers in the last 12 months.

IBJU is an unique journal because it is an open-access journal which does not charge any fee for submission and/or publication; it is produced inside SBU headquarters and every expenses are supported by the society. Furthermore, it is possible to Brazilian and other Portuguese-speaking urologists to submit their papers in Portuguese and, once approved after peer-review, get a free English version..

I was elected as Editor-in-Chief in 2012 and this is my last issue since my term will end next December. Last August, 2019, **Luciano A. Favorito, MD, PhD** was chosen to be the next Editor-In-Chief and we have been working together in the last two issues.

It has been an honor to serve SBU and to conduct this very prestigious journal. It has been a great experience. During the last eight years IBJU's impact factor has risen, the review time has decreased significantly and the journal has attracted higher quality papers.

Editorial process is a team work and I need to thank the support of the successive SBU Board of Directors, to my Associate Editors and to the group responsible for our journal production: Ricardo de **Morais, Bruno A. Nogueira** and **Patricia Gomes.**

Finally, neither peer-review journal is produced without the reviewers; in 2019, 1536 reviewers helped us voluntarily to make an even better journal. As my last act I would like to announce the five most effective reviewers of 2019, based in their review quality, number and time length of review:

**Alexandre Danilovic, MD,** Departamento de Urologia, Hospital das Clínicas da Faculdade de Medicina da USP, SP, Sao Paulo, Brasil; **David Hernandez, MD,** Department of Urology, University of South Florida, Tampa, FL, USA; **Elcio Dias Silva, MD,** Clínica Dr. Élcio Dias Silva, Campinas, SP, Brasil; **Hubert Swana, MD,** Pediatric Urology, Nemours Children's Hospital Orlando, Orlando, FL, USA; **Trushar Patel,** MD, Department of Urology, University of South Florida, Tampa, FL, USA.

See you.

